# Diabetes Mellitus across the Arabo-Islamic World: A Revolution

**DOI:** 10.1155/2023/5541808

**Published:** 2023-11-10

**Authors:** Mohamad Fleifel, Bassem Fleifel, Andrew El Alam

**Affiliations:** ^1^Endocrinology and Metabolism Division, American University of Beirut Medical Center, Beirut, Lebanon; ^2^American University of Beirut, Beirut, Lebanon; ^3^Endocrinology Division, Centre Hospitalier de Chartres, Louis Pasteur Hospital, Chartres, France

## Abstract

**Background:**

Mankind continues to suffer from the ever-growing diabetes epidemic and the rapid rise of type 2 diabetes mellitus (T2DM). This metabolic disease has been studied since ancient civilizations. The Arabo-Islamic civilization excelled in establishing some of the most notable discoveries and teachings that remained the blueprint for years to come in the field of diabetology.

**Aim:**

This article aimed to review the ancient history of diabetes mellitus, with its main focus on the Arabo-Islamic civilization, and to report our subjective views and analysis of some of the past recommendations based on modern-day findings. *Discussion*. It is natural to have the teachings of medicine dynamically inspired by one civilization to another, as various fields continue to expand and evolve. This also applies to diabetology as the Arabo-Islamic world used the outlines of prior civilizations to revolutionize the understanding of the disease. Al-Razi and Ibn Sina are probably two of the most renowned polymaths in history, and their contributions to diabetology are well documented. Ibn Maymun's postulation about the higher prevalence of diabetes in Egypt as compared to Andalusia is something to be carefully studied. It could be that diabetes mellitus' underdiagnosis and late-stage detection are some of the major reasons for the disparity between the two mentioned regions. Modern-day Arabo-Islamic scholars continue to excel in revolutionizing diabetology.

**Conclusion:**

The Arabo-Islamic world houses an impressive bout of scholars who have contributed since the ancient times to diabetology. This scientific locomotion shows no signs of stopping, as it continues to shine during the present day, and likely in the future.

## 1. Background: An Eternity of Diabetes

Diabetes mellitus is a global metabolic health problem that affects various age groups, and consequently creates a socioeconomic burden on patients and countries. Common manifestations of polyuria and polydipsia are reported as major symptoms of uncontrolled glycemia. Glycosylated hemoglobin (HbA1c) remains the current gold standard for the diagnosis of diabetes mellitus, with a value of HbA1c exceeding 6.5%, confirming the disease's existence according to the American Diabetes Association (ADA) [1]. Other diagnostic criteria include serum blood glucose (SBG) measurements, whether fasting (≥126 mg/dL (7.0 mmol/L)), random (≥200 mg/dL (11.1 mmol/L)), or through the oral glucose tolerance test (post-prandial ≥200 mg/dL (11.1 mmol/L)) [1].

**Figure 1:**
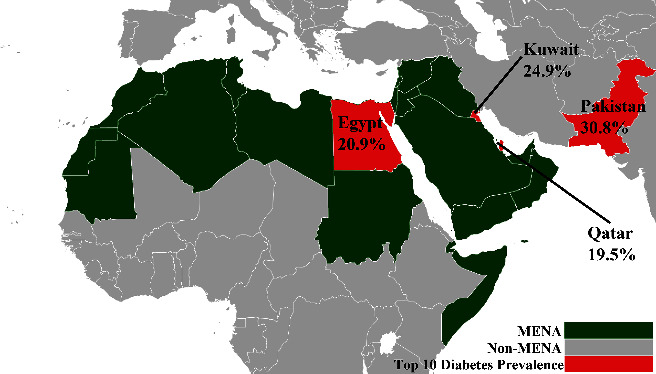
Map showing the prevalence of diabetes mellitus in three Middle Eastern and North African (MENA) countries and Pakistan. Reference: NightBag10, CC0, via Wikimedia Commons. Edited, original version available from: https://upload.wikimedia.org/wikipedia/commons/b/b3/Map_of_the_Arab_world.png.

**Figure 2:**
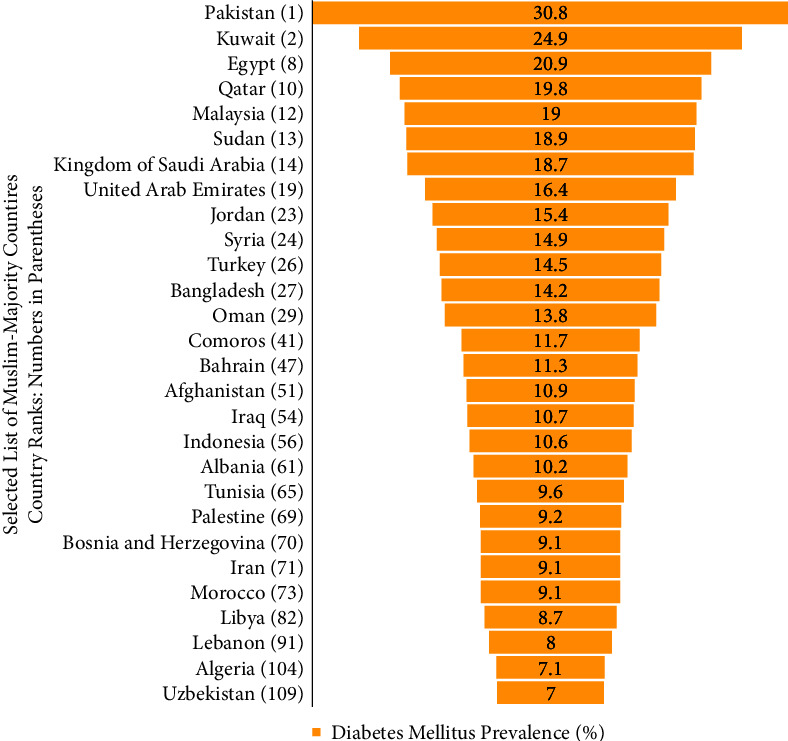
The prevalence of diabetes mellitus in selected Muslim-majority countries.

The prevalence of diabetes mellitus has been on the rise for many decades now, with type 2 diabetes mellitus (T2DM) constituting almost 90% of all diabetes prevalence according to 2021 data [2]. It is estimated that around 1.31 billion individuals worldwide could be living with the disease by 2050, with an overall global health expenditure of $1054 billion by 2045 [2]. The prevalence of diabetes has also been on the rise in the Arab World in conjunction with suboptimal nutritional habits (either due to the spread of a “Westernized diet” or the lack of appropriate nutrition), sedentary lifestyle, and subsequent overweight and obesity [3]. The Middle Eastern and North African (MENA) region has one of the rapidly increasing diabetes rates worldwide, with the number of individuals with diabetes expected to rise by 96.2% by 2035 [4]. Current world data show that 4 Muslim-majority countries, out of which 3 are in the MENA region, are ranked within the top 10 diabetes worldwide prevalence [5]. These countries are Pakistan (prevalence 30.8%; rank 1), Kuwait (prevalence 24.9%; rank 2), Egypt (prevalence 20.9%; rank 8), and Qatar (prevalence 19.5%; rank 10) (Figures 1 and 2) [5].

**Figure 3:**
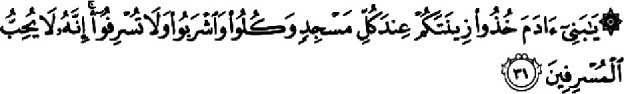
Surat Al-A'raf (Arabic for “the heights”), chapter 7, verse 31, from the holy Quran. Arabic for “O children of Adam, take your adornment at every masjid, and eat and drink, but be not excessive. Indeed, He likes not those who commit excess” (Sahih international interpretation). The verse apparently promotes healthy diet reference: the Quranic Arabic corpus—word by word grammar, syntax and morphology of the holy Quran (internet). The Quranic Arabic corpus—translation; [cited 2023 Jul 25]. Available from: https://corpus.quran.com/translation.jsp?chapter=7&verse=31.

Islam constitutes a large proportion of both Muslim majority and minority countries, as the Abrahamic religion is regarded to be one of the fastest-growing faiths in the world [6, 7]. The Islamic faith houses various diverse views regarding multiple topics, including healthcare. With religious teachings expanding on topics of illnesses and management, this might become a hindrance to healthcare providers and to patients themselves, in establishing an optimum medical care [7]. Therefore, it is essential for scholars and healthcare providers to understand Islamic beliefs in order to incorporate the best care in a culturally acceptable fashion whether that includes privacy and touch issues in physical examination, dietary regimens (e.g., during times of religious fasting), and unacceptable therapeutic plans (e.g., alcohol-based diets or medicines and overindulgence of soporific drugs) [7]. This would likely impact patient education, compliance, and the physician-patient dynamic bond. This is highly important especially in physicians communicating with Muslim patients with diabetes mellitus and the therapeutic approach to drug therapy, type of nutrition, and allowance of religious fasting. Many verses from the holy Quran and the authentic Hadith, which is a collection of traditions containing sayings as narrated by the final prophet of Islam, Muhammad–Peace Be Upon Him, refer to the importance of establishing a competent healthcare from the physician and patient's sides (Figure 3). The body and its health are visualized as a loaned commitment that the Islamic faith entrusted mankind with, thus the essentiality of caring for it [8]. Across history, multiple Islamic Arab/non-Arab and non-Islamic Arab scholars have flourished and influenced the medical field, including endocrinology and specifically diabetes. This continues to this day as more discoveries seem to rise through the coordinated efforts of these individuals. This article aimed to shed light on the topic of diabetes mellitus in the Arabo-Islamic world while detailing the journey of notable discoveries across history. We intended to review and compare such historical findings with other ancient civilizations and voiced our subjective opinions concerning the verdicts and recommendations set up by the Arabo-Islamic polymaths and scholars.

It is apparent that multiple ancient civilizations' understandings of medicine were dynamically influenced by one another. Scholars and polymaths would use the experimental methodologies and management plans of others in their academia, trials, and clinical work. Others used this knowledge to expand upon it, thus leading to revolutionary findings. Diabetes mellitus is no exception, as multiple civilizations would describe what they thought to be the standard diagnostic criteria of the disease in polyuria, polydipsia, and body wasting. We chose to briefly discuss selected ancient non-Islamic civilizations that would subsequently influence the discoveries of the Arabo-Islamic world, which was the main focus of our review (Table 1). To our understanding and knowledge, the descriptions from ancient texts referenced diabetes mellitus and more specifically T2DM, although no distinct classification was mentioned differentiating the types of diabetes mellitus.

## 2. Diabetes of the Ancient Non-Arabo-Islamic World

The Egyptian, Chinese, and Indian civilizations seemed to recognize three common main symptoms of diabetes: polyuria, glycosuria, and polydipsia with the resultant outcome of gradual body wasting, thus pointing out the catabolic nature of diabetes [9]. The description of diabetes was first documented in hieroglyphics around 1552 BCE in the now modern-day Egypt. The renowned Papyrus Ebers mentioned the manifestations of what is thought now to be uncontrolled hyperglycemia, as it documented about using plant extracts to treat patients with excessive thirst and copious urination [10]. For many scholars, this seems to be the first ever account of diabetes mellitus, although the Kahun Papyrus, the oldest known medical text (1825 BCE), highlighted the title “Treatment of a thirsty woman,” which is also suspected to describe the management of a female patient with diabetes; however, the remains of the text are still missing till date [11].

Around the 5^th^ century, Indian surgeon Sushruta coined the term “madhumeha” (Hindi for honey-like urine) as he documented the urine's sweet taste and adhesive nature in patients who developed such illness from overconsumption of carbohydrate-based diet [12]. These patients were said to suffer from polydipsia and foul breath. The Yellow Emperor's Classic of Internal Medicine text from China is thought to be the first to describe diabetes' symptoms in the Far East with it dating back to approximately 475 BCE–8 CE [13]. The term “wasting thirst” was given to the disease as it elaborated both polydipsia and the hypermetabolic state of diabetes. Zhang (150–219 CE), a well-known polymath of the Han dynasty, proposed the “three wasting-thirsts” theory: upper (linked to the lungs), middle (linked to the stomach), and lower (linked to the kidneys) [13]. Being considered a nephrological disease with its manifestation of glycosuria, it is likely that diabetes was in the “lower thirst” category. Peumery proposed that diabetes treatment should include an abstinence from wine, salt, and sexual intercourse [12]. The latter recommendation is unusual as sexual activity can be regarded as part of a physical activity which could contribute to dropping SBG. It is possible that Hsuan's recommendation, in our opinion, was based on the fear of reaching a fatigued state which could have reflected hypoglycemia, or to provide a buffer to the complaints of sexual dysfunction in uncontrolled hyperglycemia.

The Greco-Roman imprint on diabetes is there as well. Interestingly, the Hippocratic Corpus does not exhibit a frank reference to diabetes. The term “diabetes” was first used by Apollonius around 250–300 BCE, meaning siphon (i.e., to pass through) and the word “mellitus” meaning sweet [14]. Aretaeus seemed to also note that hypermetabolism associated with diabetes and revealed that people who suffered from the condition had a short and painful life [15]. Both Aulus Cornelius Celsus and Rufus of Ephesus described the polyuric nature of the disease with the latter labeling it as “urinary diarrhea” [16].

## 3. Diabetes of the Ancient Arabo-Islamic World

The Islamic golden age flourished under the Abbasid Dynasty as prominent scholars expanded upon the Indian, Chinese, and Greco-Roman medical knowledge. Probably, the earliest record of diabetes recorded in the Arabo-Islamic world is that of Al-Wathiq, the Abbasid caliph who ruled from 842 until 847 CE. It is documented that he was a victim of “insufferable thirst” which was probably a manifestation of diabetes and subsequent hepatic complications [17].

**Figure 4:**
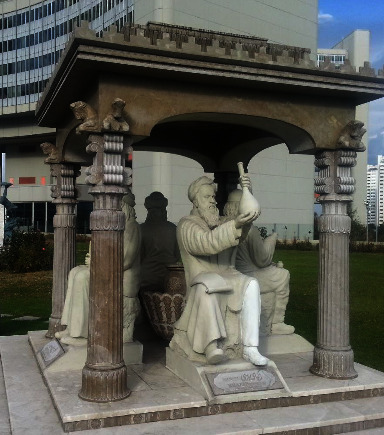
Al-Razi, aka Rhazes (middle) as part of the Scholars Pavilion or Scholars Chartagi. The monument is located outside the United Nations Office at Vienna, Austria. Credit: Yamaha5, CC BY-SA 3.0 https://creativecommons.org/licenses/by-sa/3.0, via Wikimedia Commons. Edited, original version available from: https://upload.wikimedia.org/wikipedia/commons/0/06/Persian_Scholar_pavilion_in_Viena_UN_%28Rhazes1%29.jpg.

**Figure 5:**
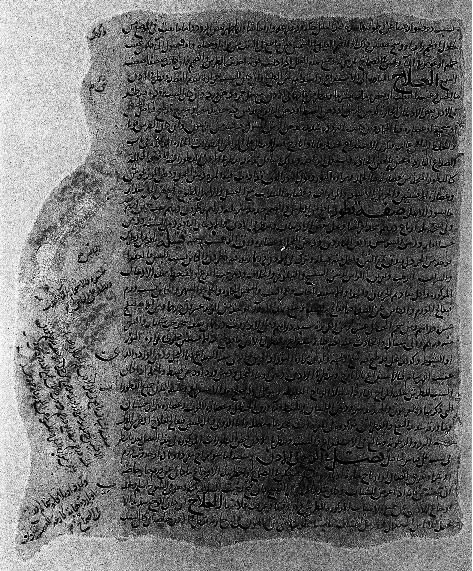
A black and white scanned copy of a medical text from Kitab al-Hawi fi al-tibb, aka The Comprehensive Book on Medicine. Credit: Arabic manuscript: Al-Hawi (Continens), Rhazes. Wellcome Collection. Public domain mark. Available from https://wellcomecollection.org/works/ymr3uxbc.

Abu Bakr Muhammad ibn Zakariya' Al-Razi (865–925 CE) (Figure 4), also known as Rhazes, was one of the most renowned physicians of the Medieval Ages, in both Islamic and non-Islamic regions. Al-Razi was the first documented Islamic scholar to write about diabetes mellitus in his many renowned textbooks, including “Kitab al-Hawi fi al-tibb” (Arabic for The Comprehensive Book on Medicine) (Figure 5) [18, 19]. Al-Razi's creative approach to diagnose diabetes included requesting patients to urinate on the sand, thus creating a nutritionally attractive environment for approaching ants [20]. If such an experiment was successful, then the patient was diagnosed with “Al-Dawwara” or “Al-Dolab” (Arabic for the rotary or the water wheel), which was the first documented Arabic name for diabetes given by Al-Razi [20]. The names reflected what was known as “Al-Na'ora” (Arabic for noria) which mirrored Apollonius' siphon description of the word diabetes. Al-Razi reported the disease's symptoms of polyuria, polydipsia, and physical wasting, therefore highlighting the severity of this chronic disease and possibly detailing diabetic cachexia. It is also postulated that he was the first to link uncontrolled hyperglycemia and visual compromise, which might be a window to diabetic retinopathy [20]. Al-Razi's description of diabetes complications, although the association was never documented, included coronary artery disease and subsequent sudden cardiac death. His near-accurate writings about clogged arteries and arrhythmia leading to subsequent syncope and cardiac arrest show his multiorgan understanding [21]. His therapy plan for diabetes included diet control, exercise, spiritual guidance, and psychological care. The latter two recommendations were exceptional, with the Al-Razi being the first physician in recorded history to include them, especially psychological care, in his management plan [20]. In his “Kitab al-Tajarib” (Arabic for The Casebook), Al-Razi detailed 41 patient cases with 4 being those of diabetes in cases II, VIII, XI, and XXIII [22].

**Figure 6:**
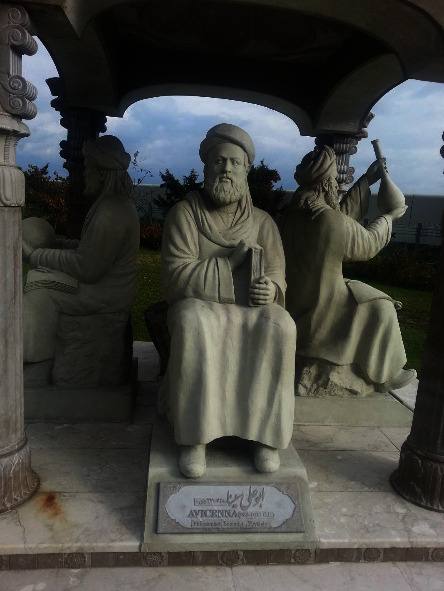
Ibn Sina, aka Avicenna (middle) as part of the scholars Pavilion or scholars Chartagi. The monument is located outside the United Nations Ooffice at Vienna, Austria. Credit: Yamaha5, CC BY-SA 3.0 <https://creativecommons.org/licenses/by-sa/3.0>, via Wikimedia commons. Available from https://upload.wikimedia.org/wikipedia/commons/f/f3/Persian_Scholar_pavilion_in_Viena_UN_%28Avicenna%29.jpg.

**Figure 7:**
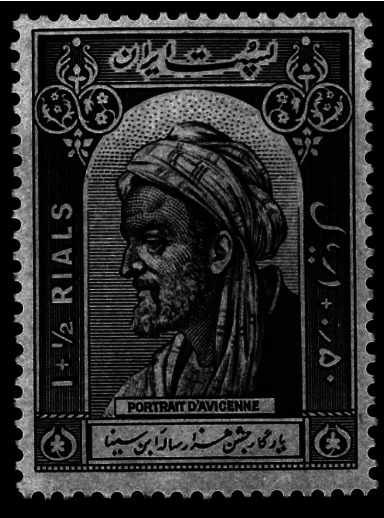
Postal stamps from Iran showing Ibn Sina. Credit: Avicenna. Postage stamps. Wellcome Collection. Attribution 4.0 international (CC BY 4.0). Edited, original version available from https://wellcomecollection.org/works/xjj3v2wh.

**Figure 8:**
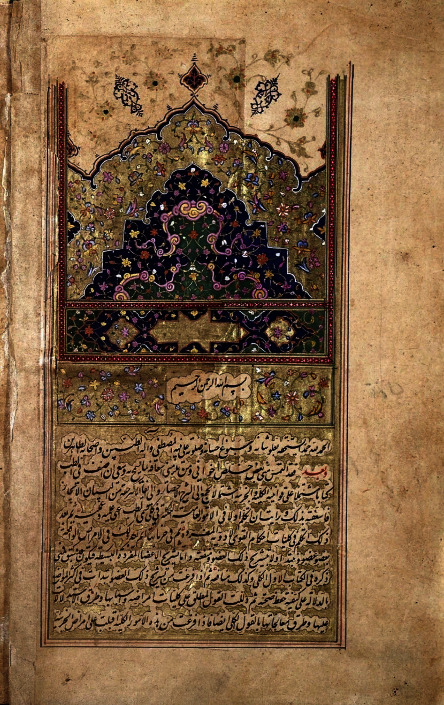
A scanned copy of a medical text from Al-Qanon Fi Al-Tib, aka The Canon of Medicine. Credit: Wellcome Collection: Avicenna, Canon Medicinae, 1632. Wellcome Collection. Attribution 4.0 international (CC BY 4.0). Available from https://wellcomecollection.org/works/bewv899r.

Ibn Sina (980–1037 CE) (Figures 6 and 7), commonly known as Avicenna, is one of the fathers of modern medicine and a pioneer polymath. His medical encyclopedia “Al-Qanun Fi Al-Tibb” (Arabic for The Canon of Medicine) is regarded as one of the finest textbooks that influenced medical teachings and training in Islamic and European regions (Figure 8) [23]. It is there where Ibn Sina had a 14-line description of diabetes' symptoms, and two pages with 22 prescription plans for treatment [24]. Around his time, the Greco-Roman-inspired terms “Ziabetes” or “Dianetes” were used to reflect the polyuric nature of diabetes, as Ibn Sina opted for either name [20, 25]. It is possible that during the transliteration from the Greco-Roman texts or dialect, the diacritic dot of the Arabic letters for “d” (د) or “b” (ࢠ) changed position to become entirely different letters in “z” (ز) or “n” (ن), respectively [24]. Like his predecessors, Ibn Sina saw diabetes as a disease of the kidneys, and he classified it into two types: Ziabetes el-barid (Arabic for cold diabetes) and Ziabetes el-har (Arabic for hot diabetes) [24, 26]. He described Ziabetes el-barid as weak kidneys which become “cold” and leads to excessive withdrawal of hepatic “moisture” to be then eliminated through the urine, creating a vicious cycle of polydipsia and polyuria. As a result, fiery heat is formed in the kidneys, creating Ziabetes el-har, which exacerbates the mentioned cycle as the kidneys attempt to cool down, therefore justifying the water wheel name in the Al-Dolab [24]. In addition to polyuria, polydipsia, and emaciation, both types were also mentioned separately in terms of symptoms: Ziabetes el-barid patients were reported to suffer from bright-colored urine, poor appetite, and poor libido. Ziabetes el-har patients had unusually colored urine, and high augmentation of libido [26]. It is possible that Ibn Sina described the symptoms of uncontrolled diabetes mellitus at different stages of chronicity and hyperglycemia as modern-day literature does not differentiate between a cold and a hot kind of disease. Ibn Sina also described the concept of Bole-e-shirin (Persian for sweet urine) which implicated the likelihood of diabetic nephropathy (DN), thus elaborating that uncontrolled glycemia and/or diabetes have been present for a significant amount of time [26]. Ibn Sina's understanding of DN pathology nearly mirrors modern-day teachings as he explained that renal exhaustion and subsequent failure could be due to a filtration problem within the “renal pores” [20]. This is similar to what current literature describes as the steps towards DN, which include hyperglycemia-induced mesangial expansion, thickening of the glomerular basement membrane, and then dilatation of the afferent renal artery and/or ischemic renal injury through hyaline narrowing of the vessels that supply the glomeruli leading to glomerular sclerosis. He highlighted the decline in sexual functions, gangrene, and muscle wasting as some of the major complications for the chronicity of the disease, as he warned of the latter manifestation which might have reflected a description of diabetic amyotrophy [27]. Ibn Sina's diabetes management included rosewater, roasted pumpkin, or sour pomegranate with water, camphor sniffing, boiled eggs (with vinegar), and diuretics [20]. The latter two are interesting as eggs are considered some of the high-protein prophetic and modern medicine options for a diabetic diet; however, others argue against egg ingestion in people at risk for diabetes or those with the disease as it might consequently lead to hypertriglyceridemia [28]. The fact that Ibn Sina recommended diuretics as one of the therapeutic remedies is impressive as current guidelines recommend sodium-glucose cotransporter-2 inhibitors (SGLT2i), which are oral antidiabetic drugs that exert their hypoglycemic and diuretic effects by inhibiting the absorption of sodium and glucose from the proximal tubule. For Ziabetes el-barid, Ibn Sina prescribed heat-generating activities which included horseback riding, hot baths, cupping, and massaging. While for Ziabetes el-har, he recommended body-cooling mechanisms including bathing in cold water, cool environmental settings, and avoiding strain on the lower back (with anesthetics being an option if needed) [24]. Al-Qanun Fi Al-Tibb remained the standard for medical teachings until the late 1700s [24, 29].

Another one of the ancient top polymaths was Abd Al-Latif Al-Baghdadi (1162–1231 CE), probably well known for his excellent philosophical work and criticism of the Arabic assimilation of Greek philosophy, who contributed to diabetology as well [30]. Al-Baghdadi's text about diabetes was made into three sections: part one discussed the clinical presentations, part two highlighted etiologies of the disease as per Greek medicine as he stated the probable hepatic and renal roles, and part three included proposed therapies [31]. His text was mainly a restatement of Ibn Sina's teachings, despite being critical of his philosophical views [30]. Ala' Al-Din Ibn Abi Al-Hazm Al-Dimashqi, commonly known as Al-Nafis (1213–1288 CE), the chief physician of the sultan Saladin-founded Bimaristan (Persian for Hospital) Al-Nasiri and the private physician for the Mamluk Sultan Al-Zahir Baybars, is most known for his discovery of the pulmonary circulation [32]. Like Al-Baghdadi, Al-Nafis' descriptions of diabetes were also similar to those of Ibn Sina, and they were covered in his book “Al-Mujiz Fi Al-Tibb” (Arabic for The Concise Book) which summarized Al-Qanun Fi Al-Tibb [24].

**Figure 9:**
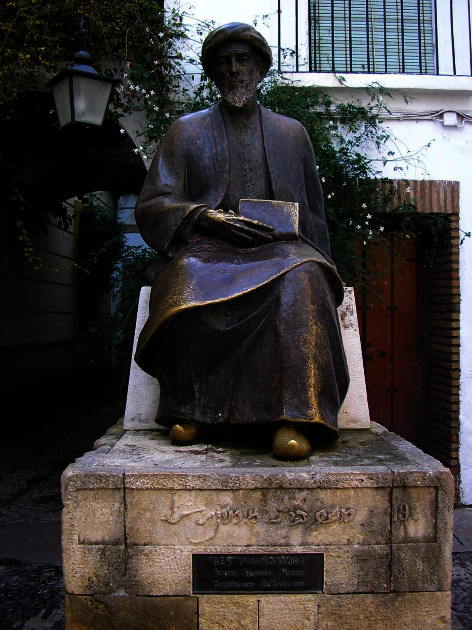
A statue of Musa Ibn Maymun, aka Maimonides, in Córdoba, Spain. Credit: Makinal∼commonswiki assumed (based on copyright claims), CC BY-SA 3.0 <https://creativecommons.org/licenses/by-sa/3.0/>, via Wikimedia commons. Available from https://upload.wikimedia.org/wikipedia/commons/2/2a/Maim%C3%B2nides.jpg.

Musa Ibn Maymun (1138–1204 CE) (Figure 9), also known as Maimonides, the famous Andalusian Jewish philosopher and personal physician for sultan Saladin, reinforced Ibn Sina's descriptions and recommendations for diabetes [24, 33]. Considering that diabetes was reportedly regarded rare, Ibn Maymun expressed his concerns over identifying 20 patients with diabetes in Egypt as compared to his native home of Andalusia and the western side of Morocco, which he contributed to the river Nile and the heat of the country [33]. A reason for such a finding could be that Egypt was the Ayyubid dynasty's origin and well-known center of prosperous economy, lavish lifestyle, and advanced medicine back then. The availability of high social-class Egyptian cuisine of sweets, creams, red meat, and fattened poultry could have probably served as a factor promoting obesity and diabetes. With Egypt's high use of trans fats, which continues to this day, makes the population susceptible to dyslipidemia, obesity, and T2DM [34]. In addition, the abundance of medical care could have prompted more diagnoses to be reported. Genetic polymorphism among Egyptians predisposing them to overweight and diabetes is also possible given what current research suggests regarding the ELMO1 gene's polymorphism as a candidate for the predilection to DN among Egyptian patients [35]. It is important to note, however, that Ibn Maymun never stated whether the diagnosed patients were native to Egypt or not. There is no current real-world evidence stating the river Nile's assumptive direct role in causing diabetes. However, a possible hypothesis could be the role of arsenic in the polluted river in leading up to diabetes. It is suggested, based on *in vitro* findings, that arsenic is involved in the dysregulation of pathways linked to pancreatic *β*-cell function and insulin sensitivity among other pathophysiological effects [36]. Egyptologists report that arsenic was used by ancient Egyptians to harden copper and as an insecticide to embalming fluid around 3,000 years ago. Public health fears were at a high when arsenic started seeping into drinking water after Nile flood waters had entered the tombs [37]. This continues to modern days as shown in a 2018 study that recorded the Nile's water quality from Qena to Sohag districts, where arsenic was detected in 36.7% of the studied samples [38]. In a Danish prospective cohort study of a mean follow-up period of 9.7 years for 52,931 individuals who had a low-level arsenic (<50 *μ*g/L) exposure in drinking water, a total of 4,304 (8.1%) had diabetes, yet the study had some reported confounders [39]. When it comes to the association between high-level arsenic exposure in drinking water and T2DM, a positive association has already been established [40, 41].

When compared to epidemiological modern-day Andalusian data, which mirror those of Ibn Maymun's time, the first national study in Spain reported a prevalence of 13.8% of T2DM, of which 6.0% had previously undiagnosed disease [42]. The incidence of known T2DM was 3.7 cases/1000 person-years in Spain, with predisposing factors including obesity, family history of T2DM, and male sex [43]. According to the PREVADIAB study, the prevalence of T2DM and pre-T2DM in Portugal was 11.7% (with 5.1% being undiagnosed) and 23.3%, respectively [44]. In a cross-sectional study evaluating the prevalence of T2DM in the adult northeastern Portuguese population, the percentage was higher than in the previously mentioned study with 17.4% and a direct association with the increase in age. This was attributed likely due to the declining per capita income compared to the European average, with the northeastern Portuguese region having a total lower per capita purchasing power than the national average [45]. When it comes to the prevalence of T2DM in Egypt, the country ranked ninth worldwide with 15.2%, with the number of adult T2DM patients being 8,850,400 in early 2020 [46]. As of 2022, Egypt stood 8th in the ranking, with a prevalence of 20.9% [5, 47]. The Sohag governorate in upper Egypt has a T2DM prevalence of 20.9%, while Alexandria houses a prevalence of 16.8% [48, 49].

Another Andalusian scholar, Abu Marwan Abd Al-Malik Ibn Zuhr (1094–1162 CE), also known as Avenzoar, famous for being the first to describe polypoid colorectal tumors and to report cases of uterine cancer and basal cell carcinoma, also had his fair share of dietary recommendations for patients with diabetes [50, 51]. His book “Al-Aghthia” (Arabic for The Nutrition) recommended multiple herbals, honey, and fish as a diabetic diet [51]. Despite his recommendations, Ibn Zuhr was reported to be fond of a high carbohydrate diet including figs and honey which consequently led to him developing diabetes. His death was likely from sepsis secondary to diabetic foot infection, a condition that was reported to have also ended the life of his father, the physician known as Abu Al-Ala' [51].

It is interesting that although the exact disease timeline (e.g., new-onset versus chronic) of diabetes diagnosis is not reported by such scholars, a likely assumption would be that since many of them reported body wasting as one of the disease's manifestations, then probably the diagnosis was either being performed in the chronic stage of the disease or in a highly uncontrolled setting of hyperglycemia. Therefore, the early stages of diabetes could have probably been underreported by the scholars back then, and the disease went undiagnosed until the late stages.

## 4. Alternative Medicine of the Arabo-Islamic World

The field of “Al-Tibb Al-Nabawi” (Arabic for Prophetic Medicine (PM)) is a form of alternative medicine which provides teachings about medical therapy as per the Islamic religious orthodox [52]. Its teachings reflect the authentic Hadith, as practiced or recommended by prophet Mohammad-Peace Be Upon Him- or Quranic Verses [53]. PM includes recommendations about healthcare whether those are certain medical regimens, nutritional habits, and social topics. It mandates the importance of an honest profession when it comes to the physician in which patients are left dignified and dedicated towards, in the perspective of Islamic law [53]. Topics could range from fever, headache, and leprosy to rules of sexual intercourse and protection from the malevolent glare (the “Ayn” which is Arabic for the evil eye). Diabetes, although not mentioned by name, is one of these topics covered by physicians who integrated the disease into PM with time.

**Figure 10:**
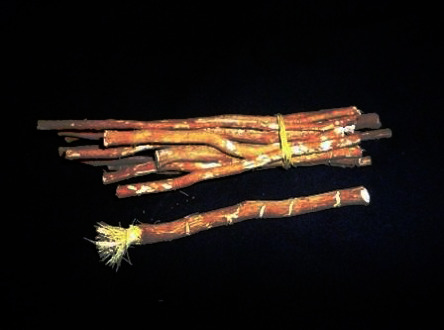
The Miswak's branches. Credit: Iqbal Osman, CC BY 2.0 <https://creativecommons.org/licenses/by/2.0>, via Wikimedia commons. Available from https://upload.wikimedia.org/wikipedia/commons/a/a2/Miswak2.jpg.

As an example of PM demonstrating proof of its efficacy, it is reported that approximately 75% of Ibn Sina's phytogenic drugs are effective in the treatment of some diabetic symptoms, with 69% possessing a hypoglycemic effect [24, 29]. Some of the commonly used PM herbals include *Cymbopogon citratus* (lemon grass), *Phoenix dactylifera* (dates palm), *Olea europaea* (olive tree), *Ficus carica* (fig), and *Salvadora persica* (miswak/mustard tree) (Figure 10), with most of them showing some evidence of antidiabetic and hypolipidemic activities [53]. The miswak's pleasant fragrance and refreshing taste have rendered its small branches a natural toothbrush since the days of the prophet. His miswak usage recommendations included pre- and post-meals, during fasting, before sleep, after rising, after entering the household, and before religious activities [54]. When it comes to its effect on metabolic diseases, multiple studies seem to highlight the antidiabetic effects of the miswak, with animal data suggesting that Arabic *S. persica* aqueous extracts at 500 mg/kg exhibited significant hypoglycemic and hypolipidemic effects, and contributed to the regeneration of pancreatic *β*-cells in streptozotocin-treated diabetic rats [55]. Another study that compared the efficacy of *S. persica* root extract to diabetic control and glibenclamide-treated rats in the reduction of the risk of diabetes concluded that, after 21 days, the hydroalcoholic extract of the *S. persica* significantly and dose-dependently exhibited hypoglycemic and hypolipidemic properties [56]. When compared to glibenclamide, the root extract did not lower SBG to the normal control levels as the sulfonylurea drug [56]. A commonly used herbal recommended by PM is the *Nigella sativa* (black cumin). *N. sativa* is an annual flowering plant of multiple bioactive compounds (including thymoquinone) that have been suggested to possess antioxidative and antidiabetic properties. It is postulated that *N. sativa* can lower HbA1c and SBG over a 60–90 days period in rodent data, with fasting SBG showing an 8 to 13% reduction [57, 58]. Based on streptozotocin-induced diabetic rats, *N. sativa* or its bioactive compound thymoquinone dosed at 400 and 50 mg/kg body weight/day, respectively, led to a fall in SBG and increased serum insulin concentration [59]. It is suggested that thymoquinone's role is the possible preservation of pancreatic *β*-cell integrity [60].

Honey is probably one of the most praised medicinal foods in the holy Quran and the Hadith [53]. It is formed from a complex mixture of sugars, with fructose and glucose being the main ones. Its novel antioxidant and antimicrobial properties have made it the subject of multiple research projects [61, 62]. A study investigating the role of low-dose honey in rats showed that honey-fed alloxan-induced diabetic rats had significantly decreased SBG levels when compared to fructose-fed healthy rats [63]. When it comes to human data, an 8-subjects study that compared the effects of dextrose solution (250 mL of water + 75 g of dextrose) to honey solution (250 mL of water + 75 g of natural honey) on SBG, plasma insulin, and plasma C-peptide, showed that honey was significantly successful in the reduction of lipids, homocysteine, and CRP in both normal and hyperlipidemic individuals, and caused a lower rise in SBG than dextrose [64]. When 30 g of natural honey was compared with 30 g of sucrose in five subjects, the former significantly promoted insulin's rise at 30, 120, and 180 minutes from consumption when compared to sucrose [64]. SBG was higher with natural honey at 30 minutes, but lower after 60, 120, and 180 minutes than that of sucrose [64]. It is also notable that, in the same study, artificial honey caused an increase in triglycerides and low-density lipoprotein-cholesterol (LDL-C) [64]. There have been multiple clinical trials that investigated the role of pure nutrition, including honey, in the treatment of T2DM while being off medical drug therapy for some period of time (20–30 days average). Most of these trials have mainly limited honey's dose to almost 30–75 g daily, and showed its hypoglycemic and lipid-lowering effects at most times, and its neutral glycemic impacts at other times without triggering any hyperglycemic emergencies (diabetic ketoacidosis or hyperglycemic hyperosmolar syndrome) [65]. Honey's exact glucose-lowering effects are still not widely understood, with a likely reason being honey's fructose ability to trigger glucokinase in hepatocytes, which leads to storage of glucose as glycogen by the liver [66]. Honey has also been used in diabetic wound healing in ancient times and up to this day [67]. Its antioxidant abilities probably help in decreasing inflammation and assisting in rapid wound healing; in addition, honey's antimicrobial capacities provide further facilitation in the healing process through its acidic pH, nitric oxide, hydrogen peroxide, and osmotic effect [65].

Vinegar is another of the recommended Islamic medicines. In streptozotocin-induced diabetic rats, after a month of vinegar administration, and compared to control animal models, the vinegar-treated rats had more weight loss, lower fasting and random SBG, higher fasting serum insulin, and higher *β*-cell proportions [68]. Based on human data, it is suggested that vinegar improves insulin sensitivity to a high carbohydrate meal in individuals with insulin resistance (34% increase during the 60-minute post-meal interval) or T2DM (19% increase during the 60-minute post-meal interval) when compared to placebo [69].

It is prudent to approach alternative medicine with caution when it comes to being the sole treatment for diabetes patients. While alternative medicine might offer some benefits, as historically recommended and documented with modern research, it is essential to be vigilant about the potential risks (e.g., hyperglycemia in sugar or carbohydrate-based products) and limitations as a sole or complementary therapy for these patients. It is vital for physicians to stay up-to-date with current worldwide-approved societal guidelines to adequately manage diabetes patients.

## 5. Diabetes of the Modern Arabo-Islamic World: The Present

In the current age, scholars, whether Arab, Muslim, or both, have been pioneers across the MENA region and the rest of the Islamic population worldwide. Dr. Ibrahim Salti, a former Deputy President of the American University of Beirut Medical Center (AUBMC), remains active to this day as a part of the formerly mentioned institute. His dedication and hard work established the first endocrinology fellowship program in the Middle East region at AUBMC [70]. Dr. Salti et al.'s work on diabetes is well-established and renowned worldwide, with the Epidemiology of Diabetes and Ramadan (EPIDAR) study serving as one of his finest works [71]. He led the discussion over the features of diabetes during Ramadan and the effect of fasting on the disease as the study included 13 countries with Muslim-based populations. Some of the important findings were that almost 43% of type 1 diabetes mellitus (T1DM) patients and 79% of T2DM patients fast during Ramadan, with an estimation of more than 50 million individuals with diabetes who fast during the holy month [71]. As of the date of our article, Dr. Mona Nasrallah serves as the chairperson and program director of the endocrinology division at AUBMC, where she has led or supervised multiple research projects [72]. Her diabetology work highlights the ever-rising prevalence of diabetes and its complications among different age groups in Lebanon and the rest of the MENA region [73–75]. One of Dr. Nasrallah et al.'s research saw an elevated incidence of T2DM in greater Beirut, the capital of Lebanon, as compared to some reported world rates, which was also similar to the high prevalence in the MENA region [76]. Dr. Sami Azar, the President-Elect of the American Association of Clinical Endocrinology-Lebanon and Middle East chapter, ranked number one in Lebanon, number 27 in Asia, and number 228 worldwide in endocrinology and metabolism according to the AD Scientific Index 2023 [77]. As part of the Lebanese chapter of the DISCOVER study, Dr. Azar and his team of coauthors described the real-world clinical practice in terms of T2DM management, which showed that metformin monotherapy was used as first-line in 56.9% and dual therapy in 25% [78].

Dr. Mahmoud Ibrahim's and Dr. Monira Al-Arouj's contributions to diabetes care in Ramadan are truly evident as they coauthored and initiated the ADA's recommendations [79]. Dr. Ibrahim et al.'s work would be updated to integrate the principles of the ADA/EASD (American Diabetes Association/European Association for the Study of Diabetes) consensus [80]. Most recently, the practical guidelines laid out by Dr. Hassanein et al., a senior endocrinology consultant in the United Arab Emirates (UAE), and his fellow authors, remain as the gold standard when it comes to diabetes care during Ramadan [81]. His work in the Canagliflozin in Ramadan Tolerance Observational Study (CRATOS) supported the use of canagliflozin in T2DM patients who fast which would be later expanded upon in the aforementioned guidelines [81, 82]. As of this article, Dr. Hassanein serves as the chairperson for the Diabetes and Ramadan (DAR) International Alliance [83].

Drs. Assam (Sam) El-Osta and Ghaith (Keith) Al-Hasani from Monash University, Australia, have led various scholarly projects in T1DM, and they have initiated the Pancreas Regeneration Project (PanRegen) [84]. Their work in attempting to restore the *β*-cell activities in T1DM has definitely been innovative; they argue that GSK126, a potent methyltransferase inhibitor and highly selective for the histone methyltransferase EZH2, can actually inhibit pancreatic EZH2 thus restoring core *β*-cell markers and ductal progenitor genes [85]. Dr. Al-Hasani has also coauthored in an article suggesting the possibility of cell reprogramming of pancreatic *α*-cells into cells displaying a *β*-cell phenotype, which could help in replenishing the body's insulin without external injections [86].

Dr. Niazi et al. shed the light on patient-centered care in Islamic communities, more specifically South Asia, with diabetes abundance [87]. He highlighted the common misconceptions laid out by the media concerning the patients' knowledge and level of education which impacts their level of understanding of the disease. His argument for the importance of the patients' informed choice, clinical education about diabetes, and enlightening significant parts of the community, such as patients' families and religious figures, could help in integrating diabetes care into a modern Muslim society. He pointed out various pieces of evidence from the holy Quran to show the untapped potential that Muslim societies have when it comes to overall adequate healthcare.

In modern Arabo-Islamic environments, addressing challenges in diabetology is of immense importance. These can be classified in relation to gender (including the reluctance of diabetes female patients in visiting male physicians), diet (examples include the use of alternative medicine instead of guidelines-based medications and patients with uncontrolled diabetes fasting during Ramadan despite the physician's recommendation against it), physical activity (for example, a decrease in outdoor activities in women and sedentary lifestyle during fasting), and misinterpretation of diabetes with respect to religious beliefs (considering diabetes as a divine test or punishment and interpreting insulin use as forbidden in regards to its assumed composition or use of needles) [87]. These challenges, of course, vary in the Arabo-Islamic world from one country to another, one local region to another, and one household to the other. With the growth in health self-awareness, people within this community seem keener on achieving what is best for their disease. The role of religious figures in issuing fatwas (i.e., rulings of an Islamic law released by a qualified authority) that decree the importance of self-care and management of current disease is vital. For patients, this would reinforce and remind them of the teachings of the Quran and the authentic Hadith about self-health care. With the rise of more diabetes literacy and numeracy, i.e., the knowledge of diabetes and ability to deal with numbers related to this condition (SBG, time intervals, and antidiabetic drug doses) respectively, in modern-day Arabo-Islamic worlds and globally, these patients are more likely to cross the line of some of the aforementioned challenges to gain the best management [88]. One important challenge to note is the socioeconomic status of many Arabo-Islamic communities, especially with most of them belonging to developing countries. The access to healthcare might be limited in some countries, especially with ongoing economic and security crises, plus the inflated prices of healthcare. This is still unfortunately ongoing with the date of this article.

Dr. Darvyri and his team from Greece (none of them part of an Arabo-Islamic environment according to our literature review) investigated the association between religiosity, spirituality, and T2DM care in a systematic review, and concluded that, regardless of the type of religious beliefs, a better glycemic control exists among those who believe in God [89]. They argued that social support, described as being part of a religious community, alleviates negative emotions and increases problem-solving skills among individuals with diabetes, which might subsequently reduce morbidity and mortality [89]. Thus, this can be explained the fact that these patients might find it easier to comprehend the seriousness of the disease and its complications, thus subsequently having a better understanding of medications' dosing and time of administration. The religion of Islam is regarded as a way of life by many who practice it; therefore, it is essential to take into consideration the patients' cultural background, including religion, to optimize on achieving an optimum therapeutic plan.

## 6. Conclusion

The human scientific discoveries and knowledge have definitely brought mankind to a level of academic and clinical enlightenment in the field of diabetology. Ancient civilizations have truly had their share of impressive findings with respect to their times, which facilitated human and animal research to mitigate our way in the diagnosis and management of diabetes mellitus. The remarkable success of the Arabo-Islamic civilization in contributing to this field has its imprint in history, and it continues to do so till todayday. This is surely something to constantly strive towards and admire the scientific revolution in this ever-growing disease.

## Figures and Tables

**Table 1:** Selected scholars from the ancient Arabo-Islamic civilization with respect to their geographical location.

Ancient Arabo-Islamic civilization		
Geographical sections	East (622–1922 CE)	West (711–1492 CE)
Countries	(i) Gulf of Arabia	(i) Andalusia
	(ii) Greater Syria	(ii) Morocco
	(iii) Iraq	(iii) Algeria
	(iv) Central Asia	(iv) Tunisia
	(v) Indonesia	(v) Mauritania
	(vi) Malaysia	(vi) Chad
	(vii) Bangladesh	(vii) Mali
	(viii) Pakistan	
	(ix) Afghanistan	

Selected scholars/polymaths	(i) Al-Razi	(i) Ibn Maymun
	(ii) Ibn Sina	(ii) Ibn Zuhr
	(iii) Al-Baghdadi	
	(iv) Al-Nafis	

## Data Availability

Our article is a narrative review. Therefore, no underlying data were collected or produced.
